# A Comprehensive Survey of Image-Based Food Recognition and Volume Estimation Methods for Dietary Assessment

**DOI:** 10.3390/healthcare9121676

**Published:** 2021-12-03

**Authors:** Ghalib Ahmed Tahir, Chu Kiong Loo

**Affiliations:** Department of Artificial Intelligence, Faculty of Computer Science and Information Technology, University of Malaya, Kuala Lumpur 50603, Malaysia; 12mscsgtahir@seecs.edu.pk or

**Keywords:** food recognition, feature extraction, automatic diet monitoring, image analysis, volume estimation, interactive segmentation, food datasets

## Abstract

Dietary studies showed that dietary problems such as obesity are associated with other chronic diseases, including hypertension, irregular blood sugar levels, and increased risk of heart attacks. The primary cause of these problems is poor lifestyle choices and unhealthy dietary habits, which are manageable using interactive mHealth apps. However, traditional dietary monitoring systems using manual food logging suffer from imprecision, underreporting, time consumption, and low adherence. Recent dietary monitoring systems tackle these challenges by automatic assessment of dietary intake through machine learning methods. This survey discusses the best-performing methodologies that have been developed so far for automatic food recognition and volume estimation. Firstly, the paper presented the rationale of visual-based methods for food recognition. Then, the core of the study is the presentation, discussion, and evaluation of these methods based on popular food image databases. In this context, this study discusses the mobile applications that are implementing these methods for automatic food logging. Our findings indicate that around 66.7% of surveyed studies use visual features from deep neural networks for food recognition. Similarly, all surveyed studies employed a variant of convolutional neural networks (CNN) for ingredient recognition due to recent research interest. Finally, this survey ends with a discussion of potential applications of food image analysis, existing research gaps, and open issues of this research area. Learning from unlabeled image datasets in an unsupervised manner, catastrophic forgetting during continual learning, and improving model transparency using explainable AI are potential areas of interest for future studies.

## 1. Introduction

Despite recent advancements in medicine, the number of people affected by chronic diseases is still large [1]. This rate is primarily due to their unhealthy lifestyles and irregular eating patterns. As a result, obesity and weight issues are becoming increasingly common around the globe. Some of the more notable diseases caused by obesity include hypertension [2], blood sugar [3], cardiovascular diseases [4], and different kinds of cancers [5]. The main reported obesity issues are in developed and middle-income countries. In 2016, 1.9 billion adults 18 years and older were overweight, while 650 million were obese. With time, children are also becoming affected by obesity at an alarming rate. According to World Health Organization (WHO), over 340 million children and adolescents between 5 and 19 years were overweight or obese [6].

The prevalence of these alarming statistics poses a serious concern. However, determining the effective remedial measures depends on different factors, ranging from a person’s genetics to their lifestyle choices. To cope with chronic weight problems, people often keep notes to track their dietary intake. In turn, dieticians require these records to estimate a patient’s nutrient consumption. However, these methods pose a challenge for users and dieticians, especially when they have to record time and estimate nutrients of diet intake [7]. For these reasons, recent research efforts have explored sophisticated vision-based methods to automate the process of food recognition and volume estimation [8,9]. The advancement in smartphone applications and hardware resources has made this more convenient, and present studies also show a higher retention rate of these mHealth apps than traditional methods [10]. Recent advancements in machine learning methods have further paved the way for more robust mHealth apps. Some dietary mobile applications such as DietLens [11], DietCam [12], Im2Calories [13], etc. integrate their apps with AI models for food recognition and ingredients detection to automate food logging. The Dietcam app also estimates nutrients from smartphone camera pictures.

However, automatic food recognition using a smartphone camera in the real world is considered a multi-dimensional problem, and the solution effectiveness depends upon several factors. Firstly, the model can achieve optimal classification performance by training with many food images for each class. Other than that, food recognition is a complex task that involves several domain-specific challenges. There is no spatial layout information that it can exploit like, in the case of the human body, the spatial relationship between body parts. The head is always present over the trunk of the human body [14,15,16] and feet towards the lower end. Similarly, the non-rigid structure of the food and intra-source variations make it even more complicated to classify food items correctly as preparation methods and cooking styles vary from region to region. Moreover, inter-class ambiguity is also a source of potential recognition problems as different food items may look very similar (e.g., soups). Moreover, in many dishes, some ingredients are concealed from view that can limit the performance of food ingredient classification models.

In addition to this, image quality from the smartphone camera is dependent on different types of cameras, lighting conditions, and orientations. As a result, the poor performance of food recognition models is highly susceptible to image distortions.

Despite these challenges, many food images possess distinctive properties to distinguish one food type from another. Firstly, the visual representations of food images are of fundamental importance as it significantly impacts classification performance. Therefore, many food-recognition methods employ handcrafted features such as shape, color, texture, and location. Recent techniques are using deep visual features for image representations. Some of these methods implement a combination of handcrafted and deep visual features for image feature representations. Secondly, for enhanced classification performance and reduced computational complexity, an appropriate selection of attributes is essential for removing redundant features from feature vectors. Finally, wisely selecting classification techniques is crucial to address food recognition challenges effectively.

Similarly, manual logging of food volume is a tedious task and involves a high rate of human error by as much as 30% [17,18,19,20,21,22]. Several solutions are proposed whose aim is to estimate food volume from smartphone camera pictures. Previous studies [23] show that using a mobile phone camera for food volume estimation increases the accuracy of the estimation of calories. Some methods involve capturing a single image, while multiple views are needed to determine accurate volume in other techniques. The food volume estimation process involves the following two steps (1) multiple images or a single image from a mobile camera is needed (2) computation of food volume from 3D construction or calibration object. Regardless of other volume estimation tasks, food volume estimation is a complex task with factors such as variations in shape and appearance due to various shapes of food and eating conditions affecting its performance.

**Figure 1:**
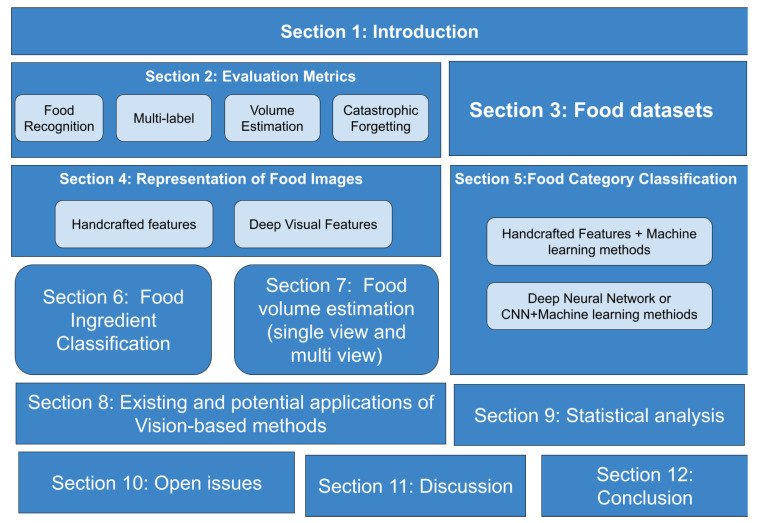
Scope and taxonomy of this survey paper.

The following research paper aims to scrutinize state-of-the-art vision-based approaches for dietary assessment to give researchers a summary of this area. Figure 1 represents the detailed scope and taxonomy of our survey study. The contribution of this survey is summarized as follows:

(1) The article briefly explores food databases for evaluating vision-based approaches and performance measures to thoroughly investigate food recognition, ingredient detection, and volume estimation methods.
(2) It presents an extensive review of food recognition techniques, including traditional methods with handcrafted features and modern deep-learning-based approaches.
(3) It provides deep insight into multi-label methods for food ingredient classification.
(4) This study surveyed most performing single-view and multi-view methods for food volume estimation.
(5) This study presents existing mobile applications that implement these approaches and other potential applications of vision-based methods in health care.
(6) The article analyzes open issues and suggests possible solutions to overcome the limitations of the existing methodologies.

It should be noted that the article is related to vision-based methods for food image analysis and their applications in the field of healthcare currently being discussed in the literature. However, the methodology of this article seeks to examine the systems more broadly by describing their important aspects similar to narrative overview [24] instead of a systematic review, some related works to the topic, or adopted search followed by a brief discussion.

Section 1 has presented the introduction of the study. The rest of the article is organized as follows. Section 2 and Section 3 examine evaluation metrics and existing datasets. Section 4 examines feature extraction methods for food image representation including handcrafted and deep visual features. In Section 5 and Section 6, we presented the most performing classifiers for food categorization and ingredient detection. Section 7 represents the food-volume-estimation methods. In Section 8, we provide brief information about mobile applications implementing these methods and other potential applications. Section 9 and Section 10 summarize statistical analysis and open issues. To conclude, we highlight our findings and future works related to this topic.

## 2. Evaluation Metrics

### 2.1. Evaluation Metrics for Food Categorization

The performance of automatic food recognition models is highly dependent on the correct mapping of food images into their respective categories. Therefore, confusion-matrix and evaluation metrics play an essential role in determining the correctness of food recognition models. Several metrics have been discussed in the literature, and their appropriate selection depends on the requirements of specific applications. It has also been observed that a classifier may perform well under one metric but poorly under another metric. For example, in the context of an imbalanced food dataset, the data samples from one or more classes outnumber data samples from the remaining food classes. Then a model trained on an imbalanced data set can have higher accuracy because of its good performance on the majority classes despite having bad classification performance on minority classes. Confusion matrix and other intrinsic metrics (Accuracy, Precision, Recall, and F1-score) generally used for detailed comparisons are discussed in detail below.

#### 2.1.1. Confusion Matrix

**Figure 2:**
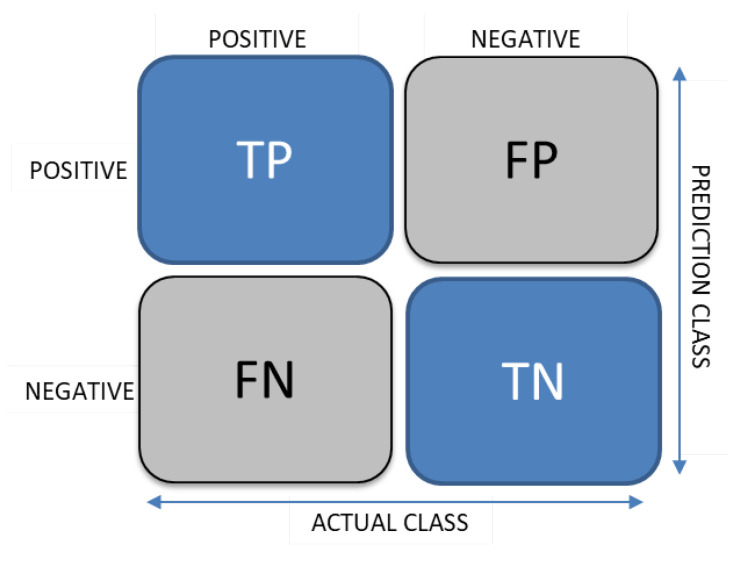
Confusion matrix.

Confusion matrices are a widely used approach to summarize the performance of a classification model in machine learning. In some cases, classification accuracy alone can be misleading, especially when there are more than two classes in a dataset or if there were an unequal number of observations present in food classes. Therefore, the confusion matrix provides a clear picture of actual and predicted classes obtained by the classification model. The confusion matrix is basically a two-dimensional matrix where each row represents an example of an actual food class and each column represents a state of the predicted food class. TP stands for true positive, TN represents the number of true negatives, FP is the number of false positives, and FN represents false negatives in the confusion matrix shown in Figure 2.

#### 2.1.2. Accuracy

The accuracy of a model determines whether the model is able to predict food classes correctly or how well a certain model can generally perform. Equation (1) represents the mathematical form of accuracy. However, accuracy cannot be used as a major performance metric, as it does not serve the purpose when there is an imbalanced dataset. Therefore, we have incorporated Precision, Recall, and F1 score to provide better insights into the results.

(1) $$\mathrm{Accuracy}\;=\frac{\;(TP+FN)}{\left(TP+FP+FN+TN\right)}\times 100\;$$

Here TP refers to the true positive. True positive is an outcome where the model has correctly predicted a positive class. For example, in the case of food recognition, it refers to the food class that the model is trying to predict. TN refers to the true negatives: the prediction is correct, and the actual value is negative. In the case of food recognition, it refers to images from those food classes that the model is not trying to predict. FP refers to the false positive, and FP prediction results are wrong. For example, in the case of Food/NonFood recognition, FP refers to images that are non-food but are predicted as food. FN refers to the false negatives. It refers to those data samples which are positive but wrongly classified as negative class. For example, those food images that are classified as non-food images by model.

#### 2.1.3. Precision

The Precision score can be defined as how often a model can correctly predict values classified as positives. In simpler words, out of all predicted positive food classes, it indicates what percentage is truly positive. This score is beneficial when the cost of false positives is high. It is calculated by Equation (2).

(2) $$\mathrm{Precision}\;\mathrm{Score}\;=\frac{TP}{\left(TP\;+FP\right)}\;\;$$

#### 2.1.4. Recall

Recall score identifies the model’s ability to correctly classify food classes. It determines out of total positive food classes what percentage is predicted positives. It provides better insight when the cost of false negatives is high. It is computed by using Equation (3).

(3) $$\mathrm{Recall}\;=\;\frac{TP}{\left(TP+FN\right)}$$

#### 2.1.5. F1 Score

F1 score represents the harmonic mean of recall and precision score. It considers both false positives and false negatives; therefore, it performs great on imbalanced datasets. It is calculated by following Equation (4).

(4) $$F1\;\mathrm{Score}\;=\;\frac{\left(2*(\mathrm{Precision}*\mathrm{Recall})\right)}{\mathrm{Precision}+\mathrm{Recall}}\;$$

### 2.2. Catastrophic Forgetting during Progressive Learning

Food datasets are open-ended due to the large variety of food dishes and different preparation styles. There are no limitations and constraints on the number of classes, and the model can progressively adapt domain variations in existing classes while learning new food classes. However, catastrophic forgetting during progressive learning causes the neural network to forget previous knowledge while learning new concepts. Catastrophic forgetting measures compute the algorithm’s ability to retain previous concepts and knowledge while learning new information. Kemker et al. [25] and Chaudry et al. [26] proposed five measures of catastrophic forgetting to achieve this objective.

#### 2.2.1. Intransigence

This refers to the difference in classification performance between the reference model trained by batch learning technique and the model trained on feature vectors using incremental learning protocol. The negative intransigence shows that incrementally learning a new set of food classes improves performance. Equation (5) denotes its mathematical form.

(5) $$l_{k}=a_{k}^{*}-a_{k}{,}_{k}$$

#### 2.2.2. Forgetting

This refers to the difference between the highest classification performance of a particular session in previous sessions and its classification performance in the current sessions. Equation (6) computes the average forgetting of the network up to the *k*th session.

(6) $$\begin{array}{c} f_{j}^{k}=max_{1\in \{1,\cdots \cdots ,K-1\}}\;a_{i,j}-a_{\left(k,j\right)},j>k\\ F_{k}=\frac{1}{k-1}\sum_{j=1}^{k-1}f_{j}^{k}\; \end{array}$$

#### 2.2.3. Base Session

This refers to the model’s ability to retain the knowledge of base food classes in current sessions, as shown in Equation (7).

(7) $$\Omega_{base}=\frac{1\;}{k-1}\sum_{j=2}^{k}\frac{a_{j,1}}{a_{ideal}}\;$$

#### 2.2.4. New Session

This is the ability of a model to recall newly learned food classes, as shown in Equation (8).

(8) $$\Omega_{new}=\frac{1\;}{k-1}\sum_{j=2}^{k}a_{j,j}\;$$

#### 2.2.5. All Session

This refers to the retention of the previous food classes learned by the network when learning new food classes, as computed by Equation (9).

(9) $$\Omega_{all}=\frac{1\;}{k-1}\sum_{j=2}^{k}\frac{a_{j,all}}{a_{ideal}}\;$$

### 2.3. Evaluation Metrics for Food Ingredient Classification

Similarly, food ingredient recognition is equally important for dietary assessment applications. As food categorization is limited to the classification of generic food items present in the food images, food ingredient recognition and classification provide deep insights into the caloric content present in the food image. Therefore, food ingredient recognition applications widely incorporate multi-label classification [27]. Since food ingredient recognition is considered a multi-label problem as food images usually contain more than one ingredient. Therefore, evaluation metrics generally used for multi-label classification are different from traditional single-label classification. The following are the performance metrics are used by food ingredient recognition models.

Consider $x_{i},Y_{i}\;$ with *L* number of labels as training datasets. Let us assume that MLC is the training method and $Z_{i}=MLC\left(x_{i}\right)\;$ is the output labels (ingredients) predicted by the classification method.

#### 2.3.1. Precision

Precision is the ratio of correctly predicted labels to the total number of actual labels, averaged across all instances. Equation (10) represents precision for food ingredient classification.

(10) $$\mathrm{Precision}\;=\;\frac{1}{N}\sum_{i=1}^{N}\;\left(\frac{MLC\left(x_{i}\right)\;\cap Y_{i}}{MLC(x_{i})}\right)$$

#### 2.3.2. Recall

Recall is computed by Equation (11). It is the ratio of correctly predicted labels to the total number of predicted labels.

(11) $$\mathrm{Recall}\;=\;\frac{1}{N}\sum_{i=1}^{N}\;\left(\frac{MLC\left(x_{i}\right)\;\cap Y_{i}}{MLC(Y_{i})}\right)$$

#### 2.3.3. F1 Score

Finally, F1 score is the harmonic mean of the precision and recall. Equation (12) represents the F1 score.

(12) $$F1\;\mathrm{Score}\;=\;\frac{1}{N}\sum_{i=1}^{N}\;\left(\frac{2*\;\left|MLC\left(x_{i}\right)\;\cap Y_{i}\right|}{\left|MLC(x_{i})\right|\;+\;\left|Y_{i}\right|}\right)$$

### 2.4. Evaluation Metrics for Food Volume Estimation

Similarly, various studies related to food volume estimation use ground truth values to compare the accuracy of their proposed methods to determine the accurate food volume [28,29,30,31,32,33,34,35,36,37,38,39]. Unfortunately, there is no dataset available to date for accurate measurement of food volume. Nevertheless, the method proposed by [40] uses controlled experiments that require participants to click images before and after their meal to compute consumed calories, which are later compared with ground truth values. Similarly, Ref. [41] incorporated different food models to determine the true volume; however, various models failed to provide accurate information. Therefore, they implemented the water displacement method, which requires a mean of three readings to find out the true volume. Furthermore, most studies used the following equations to compute the relative error and estimate the accuracy of the method

(13) $$e\;=\;\left|v\;-\;v_{approx}\right|$$

where *v* is the actual volume and $v_{approx}$ is the approximate volume

(14) $$e\;=\;\frac{1}{N}\sum_{i=1}^{n}\frac{\left|w_{i\;-}\;w_{g}\right|}{w_{g}}$$

where *N* is the number of food items, $w_{i}$ is the estimated weight of the food item, and $w_{g}$ is the ground truth value of the food.

## 3. Datasets Used for Food Recognition

Performance of feature extraction and classification techniques is highly dependent on the detail-oriented collection of images, which, in our case, happen to be food images. As consolidated large food image datasets, for example, UECFOOD-100, Food-101, UECFOOD-256, UNCIT-FD1200, and UNCIT-FD889 are eventually used as benchmarks to collate recognition performance of existing approaches with new classifiers. Such datasets can be distinctive in terms of characteristics, such as the total number of images in a particular dataset, cuisine type, and included food categories.

For instance, UECFOOD-100 contains 100 different sorts of food categories, and each food category has a bounding box that indicates the location of the food item in the photograph. Food categories in this dataset mainly belong to popular foods in Japan [42]. Similarly, UECFOOD-256 is another variant of UECFOOD-100. However, it differs in terms of the number of images as it contains 256 food images of different kinds [42]. Food-101 contains 101,000 real-world images that are classified into 101 food categories. It includes diverse yet visually similar food classes [43]. Similarly, the PFID food dataset is composed of 1098 food images from 61 different categories. The PFID collection currently has three instances of 101 fast foods [44]. UNCIT-FD1200 is composed of 4754 food images of 1200 types of dishes captured from actual meals. Each food plate is acquired multiple times, and the overall dataset presents both geometric and photometric variability. Similarly, UNICT-FD 889 dataset has 3583 images [45] of 889 different real food plates captured using mobile devices in uncontrolled scenarios (e.g., different backgrounds and light environmental conditions). Moreover, they capture each dish image in UNICT-FD899 multiple times to ensure geometric and photometric variability (changes in rotation, scale, and point of view) [46].

**Figure 3:**
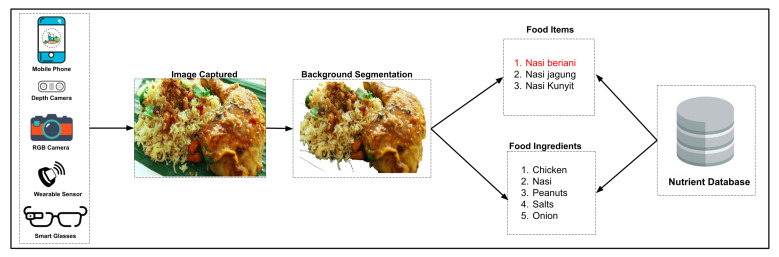
System Flow.

**Figure 4:**
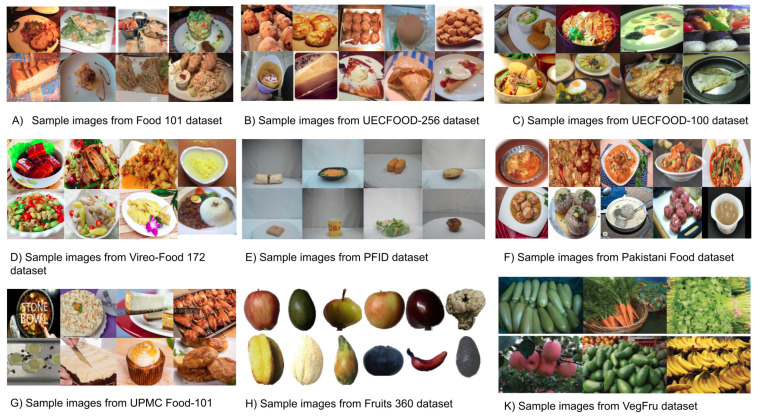
Sample images from few food datasets.

Several datasets mainly consist of various food images collected through various sources such as web crawlers and social media platforms such as Instagram, Flickr, and Facebook. Furthermore, most of these datasets contain images of foods that are specific to certain regions, such as Vireo-Food 172 [47] and ChineseFoodNet [48]. Both datasets contain Chinese dishes. Similarly, Food-50 [49], Food-85 [49], Food log [50], UECFOOD-100 [42], and UECFOOD-256 [43] contain Japanese Foods items. Turkish foods-15 [51] is limited to Turkish food items only. Furthermore, the Pakistani Food Dataset [52] accommodates Pakistani dishes, and the Indian Food Database incorporates Indian cuisines. In addition to this, few datasets only include fruits and vegetables like VegFru [53], Fruits 360 Dataset [54], and FruitVeg-81 [55]. Furthermore, Table 1 provides a brief description about food image datasets. Figure 3 shows the system flow and Figure 4 shows the sample images from the food datasets.

Therefore, it is evident from the survey that there is an immense need for broad and generic food datasets for better food recognition and enhanced performance. This necessity is because region-specific food items or datasets with fewer food categories can undermine the accuracy and performance of classification and extraction methods.

## 4. Representation of Food Images

Feature extraction plays a vital role in automated food recognition applications due to its noticeable impact on the recognition efficiency of an employed system. Feature extractors methods extract different food image representations. The process of feature extraction involves the identification of visual characteristics like color, shape, and texture. The main objective of feature extraction is to reduce dimensionality space [79] and extract more manageable groups from raw vectors of food images.

Moreover, selecting the right set of features ensures that relevant information is extracted from input images to perform the desired task. We categorized the feature extraction techniques into two main types: hand-crafted and deep visual features. The term ‘handcrafted’ refers to identifying relevant feature vectors of appropriate objects such as shape, color, and texture. In contrast to that, the deep model provides state-of-the-art performance due to automatic feature extraction through a series of connected layers. For this reason, recent studies have adopted combinations of both hand-crafted and deep visual features for food image representation.

### 4.1. Handcrafted Features

**Figure 5:**
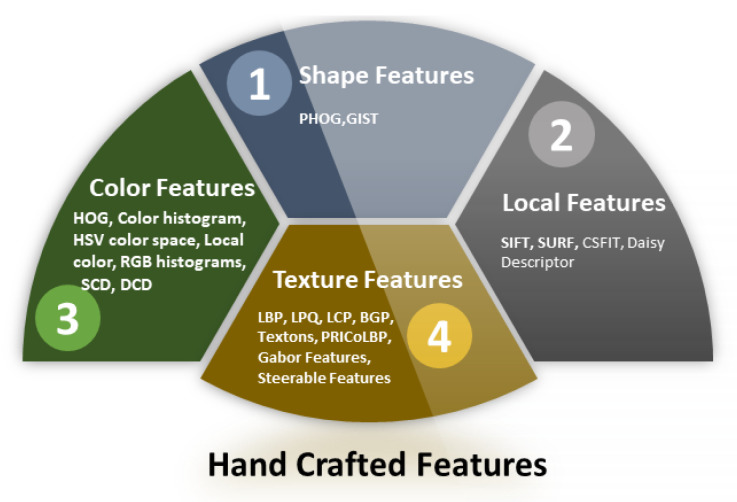
Handcrafted feature extraction methods.

The existing literature exhibits a large number of methods to employ manually designed or handcrafted features. Handcrafted features are properties obtained through algorithms using help from information available in the image. Figure 5 categorizes the handcrafted feature extraction methods. In the scenario of food image recognition, there is variation among different food types in terms of texture, shape, and color.

The term ‘texture’ refers to homogeneous visual patterns that do not result from single colors such as sky and water [7]. Textural features usually consist of regularity, coarseness, and/or frequency. Texture-based characteristics are classified into two classes, namely statistical and transform-based models. Similarly, shape features attempt to quantify shape in ways that agree with human intuition or aid in perception based on relative proximity to well-known shapes. Based on the analysis, these shapes can be declared either perceptually similar to human perception or different. Furthermore, extracted features should remain consistent concerning rotation, location, and scaling (changing the object size) of an image. Unlike shape and texture features, color features are prevalent for image retrieval and classification because of their invariant properties concerning image translation, scaling, and rotation. The key items of the color feature-extraction process are color quantization and color space. Therefore, the resulting histogram is only discriminative when it projects the input image is to the appropriate color space. Different methods are widely employed for food classification, including hue, saturation, value (HSV); CIELab; red, green, and blue (RGB); normalized RGB; opponent color spaces; color k-means clustering; bag of color features; color patches; and color-based kernel. Although the color features from the food images distinguish between different food items, due to intra-class similarity, these features alone are not enough to accurately classify food images. For this reason, most researchers have used color features in combination with other feature extraction methods.

Hoashi et al. [49] employed bag of features, color histogram, Gabor features, and gradient histogram with multiple kernel learning for automatic food recognition of 85 different food categories. Similarly, Yang et al. [80] dealt with pairwise statistics between local features for food recognition purposes using the PFID dataset. For real-time food image recognition, Kawano and Yanai et al., 2014 [43] utilized handcrafted features such as color, histogram of oriented gradient (HoG), and Fisher Vector (FV). Moreover, the cloud-based food recognition method proposed by Pouladzadeh et al., 2015 [81], involves features like color, texture, size, shape, and Gabor filter. They evaluated their framework on single food portions consisting of fruit and a single item of food. Furthermore, mobile food recognition systems proposed by Kawano and Yanai, 2013 [82], and Oliveira et al., 2014 [83], also used handcrafted features like color and texture. Table 2 summarizes the details of proposed methods that employ handcrafted features for food recognition.

However, identification of food involves challenges due to varying recipes and presentation styles used to prepare food all around the globe, resulting in different feature sets [84]. For instance, the shape and texture of a salad containing vegetables differ from the shape and texture of a salad containing fruits. For this reason, we should optimize the feature extraction process by extracting relevant visual information from food images. Such data are present in general information descriptors, which are a collection of visual descriptors that provide information about primary features like shape, color, texture, and so forth. Some important descriptors used in existing studies include Gabor Filter, Local Binary Patterns (LBP), Scale-invariant Feature Transform (SIFT), and color information to extract features of food images [85]. These descriptors can be applied individually or in combination with other descriptors for enhanced accuracy.

Nonetheless, feature selection remains a complex task for food types that involve mixed and prepared foods. Such food items are difficult to identify and are not easily separable due to the proximity of ingredients in terms of color and texture features. In contrast, the evolution of deep learning methods has remarkably reduced the use of handcrafted features. This is due to their superior performance for both food categorization and ingredient detection tasks. However, handcrafted methods for feature extraction may still serve as the foundation for automated food recognition systems in the future.

### 4.2. Deep Visual Features

Recently, deep learning techniques have gained immense attention due to their superior performance for image recognition and classification. The deep learning approach is a sub-type of machine learning, and it trains more constructive neural networks. The vital operation of deep learning approaches includes automatic feature extraction through the sequence of connected layers leading up to a fully connected layer, which is eventually responsible for classification. Moreover, in contrast to conventional methods, deep learning techniques show outstanding performance while processing large datasets and have excellent classification potential [93,94].

Deep learning methods such as Convolutional Neural Networks (CNNs) [95], Deep Convolutional Neural Networks (DCNNs) [96], Inception-v3 [97], and Ensemble net are implemented by existing food recognition methods for feature extraction. Convolutional Neural Networks are one of the widely used deep learning techniques in the area of computer vision due to their impressive learning ability regarding visual data, and they achieve higher accuracy than other conventional techniques [98]. The DCNN technique gained popularity owing to its large-scale object recognition ability. It incorporates all major object recognition procedures such as feature extraction, coding, and learning. Therefore, DCNN is an adaptive approach for estimating adequate feature representation for datasets [99]. Similarly, Inception-v3 is also a new deep convolutional neural network technique introduced by Google. It is composed of small inception modules that are capable of producing very deep networks. As a result, this model has proved to have higher accuracy, decreased number of parameters, and computational cost in contrast to other existing models. Likewise, Ensemble Net is a deep CNN-based architecture and is a suitable method for extracting features. It is due to the outstanding performance of CNN feature descriptors as compared to handcrafted features.

Asymmetric multi-task CNN and spatial pyramid CNN [100] provides highly discriminative image representations. Jing et al. [47] proposed ARCH-D architecture for multi-class multilabel food recognition, and their model provides feature vectors for both food category and ingredient recognition. Although the feature vectors from multi-scale multi-view deep network [101] has a very high dimension, they were successful in achieving state-of-art performance. Ghalib et al. [52] proposed ARCIKELM for open-ended learning. They have employed InceptionResnetV2 for feature extraction due to their superior performance over other deep feature extraction methods such as ResNet-50 and DenseNet201. Table 3 further provides a brief description of deep visual features.

## 5. Food Category Classification

The primary requirement of any food recognition system is accurate identification and recognition of food components in the meal. Therefore, robust and precise food classification methods are crucial for several health-related applications such as automated dietary assessment, calorie estimation, and food journals. Image classification refers to a machine learning technique that associates a set of unspecified objects with a subset (class) learned by the classifier during the training phase. In the scenario of food image classification, food images are used as input data to train the classifier. Hence, an ideal classifier must recognize any food category explicitly included during the learning phase. The accuracy of a classifier mainly depends on the quantity and quality of images, as there are several variations in food images such as rotation, distortion, lightning distribution, and so forth. In this section, we discuss classification techniques used by traditional approaches that use handcrafted features. Following that, we analyzed state-of-the-art deep learning models for food recognition.

### 5.1. Traditional Machine Learning Methods

Major classifiers used by several traditional approaches in the domain of food image recognition include Support Vector Machines (SVM) [49], Multiple Kernel Learning (MKL) [49] and K-Nearest Neighbor (KNN) [47]. It is due to their outstanding performance as compared to other classification methods.

The food recognition method proposed by [121] employs color, SIFT, and texture features to train the KNN classifier. In contrast to SVM, KNN achieved higher classification accuracy, i.e., 70%, whereas the accuracy of the SVM classifier was only 57%. Similarly, treatment of diabetic patients involves a daily insulin prandial dose to compensate for the effect of a meal, and its estimation is a complex task with carbohydrate counting being a key element. To assist patients in automating the process of counting CHO from images captured from a camera, Anthimopoulos et al. [89] applied a bag-of-features model using SIFT features. A linear SVM classifier trained on food images of 11 different food classes acquired a classification accuracy of 78%.

Chen et al. [48], employed a multi-class SVM classifier for the identification of 50 different classes of Chinese food. It includes 100 food images in each category. However, classification accuracy was only 62.7%. They further implemented a multi-class Adaboost algorithm and increased their classification accuracy up to 68.3%. Furthermore, Bejibom et al. [64] used LBP, color, SIFT, MR8, and HoG features to train an SVM image classifier. They evaluated their work on two different datasets and achieved a classification accuracy of 77.4% on the dataset presented by [48]; their classification accuracy was 51.2% when applied to the menu-matched dataset. Table 4 summarizes classifiers implemented by traditional classification methods along with their achieved classification accuracies.

### 5.2. Deep Learning Models

Deep learning approaches have gained significant attention in the field of food recognition. This is due to their exceptional classification performance in comparison to traditional approaches [48,64]. convolutional neural network (CNN), deep convolutional neural network (DCNN), Ensemble Net, and Inception-v3 are some of the most prominent techniques used as existing methods for food image recognition purposes.

Yanai and Kawano [102] employed a deep convolutional neural network (DCNN) on three food datasets: Food-101, UECFOOD-256, and UECFOOD-100. They explored the effectiveness of pre-training and fine-tuning a DCNN model using 100 images from each food category obtained from each dataset. During evaluation, classification accuracy achieved was 78.77% for UECFOOD-100, 67.57% for UECFOOD-256, and 70.4% for Food-101. Similarly, the study presented by [105] implemented Inception-v3 deep network established by Google [97] on the same datasets, i.e., Food-101, UEC FOOD-100, and UECFOOD-256. Classification accuracy achieved using fine-tuned model V3 was greater than classification accuracy of the fine-tuned version of DCNN i.e., 88.28%, 81.45%, and 76.17% for UECFOOD-100, UECFOOD-256, and Food-101, respectively. The food recognition method proposed by [106] implemented a CNN-based approach using the Inception model on the same three datasets.

Classification accuracy achieved was 77.4%, 76.3% and 54.7% for UECFOOD-100, UECFOOD-256 and Food-101, respectively. Table 5 provides the overview of existing food recognition methods based on deep learning approaches and their classification performance.

## 6. Food Ingredient Classification

Over the past few years, nutritional awareness among people has increased due to their intolerance towards certain types of food, mild or severe obesity problems, or simply interest in maintaining a healthy diet. This rise in nutritional awareness has also caused a shift in the technological domain, as several mobile applications facilitate people in keeping track of their diet. However, such applications hardly offer features for automated food ingredient recognition.

For this purpose, several proposed models use multi-label learning for food ingredient recognition. It can be defined [27] as the prediction of more than one output category for each input sample. Therefore, food ingredient recognition is known as a multi-label learning problem. Marc Bolanos et al. have deployed CNN as a multi-label predictor to discover recipes in terms of the list of ingredients from food images [131]. Similarly, Yunan Wang et al. [132] used multi-label learning for mixed dish recognition, as they have no distinctive boundaries among them. Therefore, labeling bounding boxes for each dish is a challenging task. Another system proposed by Amaia Salvador et al. [133] regenerates recipes from provided food images along with cooking instructions. On the other hand, Jingjing Chen and Chong-Wah Ngo [47] proposed deep architectures for food ingredient recognition and food categorization and evaluated their proposed system on a large Chinese food dataset with highly complex food images. Food ingredient recognition is often overlooked and is a challenging task, as it requires training samples under different cooking and cutting methods for robust recognition. Therefore, methods proposed by Chen et al. [134] and J. Chen et al. [135] focus on food ingredient recognition. The authors Chen et al. [134] deploy multi-relational graph convolutional network that was later evaluated on Chinese and Japanese food datasets, resulting in 36.7% for UECFOOD-100 and 48.8% for VireoFood-172. However, Chen et al. [135] proposed DCNN based method for food ingredient recognition and achieved Top 1 accuracy up to 86.91% and Top 5 accuracy up to 97.59% for Vireo Food-251.

Furthermore, Table 6 provides brief information about accuracy scores of proposed systems along with methods and dataset used.

## 7. Food Volume Estimation

**Figure 6:**
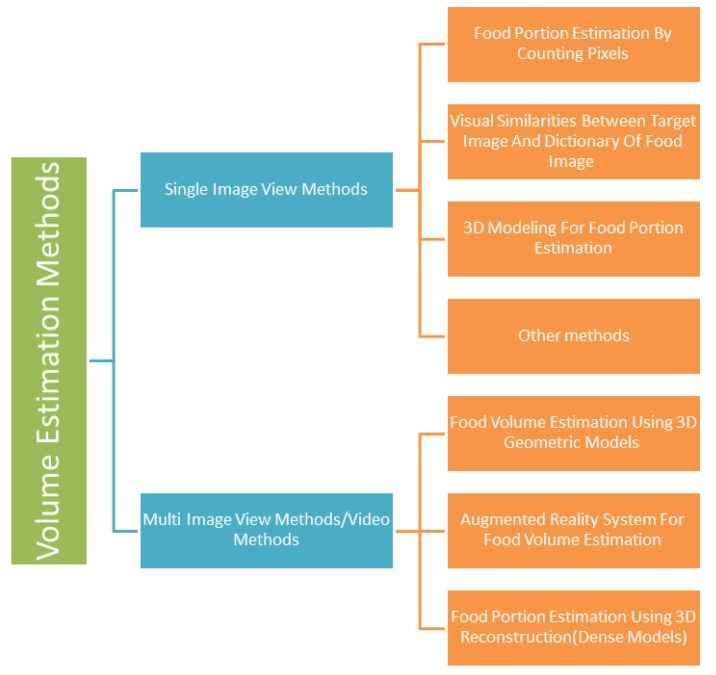
Food Volume Estimation Methods.

Automated food volume assessment is a convoluted task involving various challenges. Highly diverse and varying compositions of food, increasing varieties of ingredients, and different methods of preparations are only some of the factors that need to be taken into consideration. Furthermore, the quality of pictures taken for food volume estimation also impacts the accuracy. Clear pictures taken in good lighting conditions would yield different results compared to low-resolution or low-light images. Thus, far, several methods have been proposed for accurate estimation of food volume ranging from simple techniques such as pixel counting to complex methods such as 3D image reconstruction. They have been broadly categorized as either ‘single image view’ or ‘multi-image/video view’ methods in the subsequent sections. Figure 6 shows the types of food volume estimation methods.

### 7.1. Single Image View Methods

Single-Image-View Methods for food volume estimation require only a single image for food volume estimation. These methods are relatively more user-friendly than ‘multi-image view methods’ because they do not require multiple images from different viewpoints. However, as a trade-off, most of the single-view methods are less accurate in contrast to multi-view methods. Table 7 summarizes single view methods for volume estimation. The following are a few common methods that use the single-view method for food portion estimation:

#### 7.1.1. Food Portion Estimation by Counting Pixels

This method utilizes pixel count in each relevant image section to estimate food portion size. Studies [120] show that these methods are less complex than methods that rely on 3D modeling. Despite its simplicity, it gives a good estimation of portion size, thus making calculation of caloric content and nutritional facts easier.

#### 7.1.2. Visual Similarities between Target Image and Dictionary of Food Images

This method estimates visual similarities between a given image and an existing food image dictionary. It is used by many existing systems today [29], where the caloric and nutrient contents in the food image dictionary are defined by dietary professionals to get a better approximation. The method selects first ‘n’ images from the dictionary and calculates the calorie content of the target image based on the average calorie content of dictionary images.

#### 7.1.3. 3D Modeling for Food Portion Estimation

This method projects a 3D model of food portions onto 2D space or uses 3D geometric models for volume estimation. Generally, this method gives finer approximation in contrast to the other methods for single-image-view methods.

#### 7.1.4. Other Methods

Other methods for food-portion estimation include estimating portion sizes using a ruler and adjustable wedge [56], mobile augmented reality, virtual reality [33], visual assessment [137] feature extraction, and its matching [29,64].

### 7.2. Multi-Image View or Video Methods

Multi-Image view or video methods require multiple images for food portion estimation. They are relatively more accurate than single-view-image methods. However, multi-image methods are less user-friendly as they require multiple images from different viewpoints in order to provide better results. Table 8 summarizes single-view methods for volume estimation. The following are a few methods that use multi-image-view techniques for food volume estimation.

#### 7.2.1. Food Volume Estimation Using 3D Geometric Models

This multi-image-view method uses a shape template method or 3D modeling for portion size estimation. As a single shape template is not suitable for all food types, the use of geometric models with correct food classification labels and segmentation masks in the image is important to index food labels to their respective classes of predefined geometric models. These can be used later for finding correct parameters of the selected geometric model [28,40,41,56,62].

Moreover, in 3D modeling and pose estimation, models for food are constructed in advance by using between 15 and 20 food images captured from several angles or a video sequence. Finally, food volume is estimated by registering pose from 3D models to 2D images [36].

#### 7.2.2. Augmented Reality System for Food Volume Estimation

The use of augmented reality is also being widely used by researchers to estimate food portion size. Many systems such as Eat AR make use of it for portion size estimation [60] by developing prototypes to aid users. These prototypes generally require fiducial markers or credit-card-sized objects for overlaying 3D forms. Finally, the volume of the overlaid forms is computed using a signed volume estimation algorithm for closed 3D objects.

Similarly, the ‘Serv Ar’ augmented reality tool is used to provide guidance about food serving size [147]. Many of these technologies are being used with object recognition methods to identify food items and determine their caloric content. Similarly, methods that use augmented reality in combination with other portion estimation techniques have enhanced accuracy and much more interactive interfaces, resulting in a high retention rate.

#### 7.2.3. Food Portion Estimation Using 3D Reconstruction (Dense Models)

Portion estimation by constructing dense 3D models usually requires multiple images or a video segment [139]. Joachim Dehais et al. [148] have shown the use of two views for volume estimation using 3D construction. In its first stage, the system learns about the configuration of different views, followed by the construction of a dense 3D model to extract the volume of each individual food item placed before it. Similarly, Wen Wu et al. [32] studied the use of fast food videos for caloric estimation. Most of these methods require images from different viewpoints, and for this reason, more advanced methods such as 3D construction from accidental motion can be explored for food volume estimation in the future.

### 7.3. Strengths and Weakness of the Food Volume Estimation Methods

**Figure 7:**
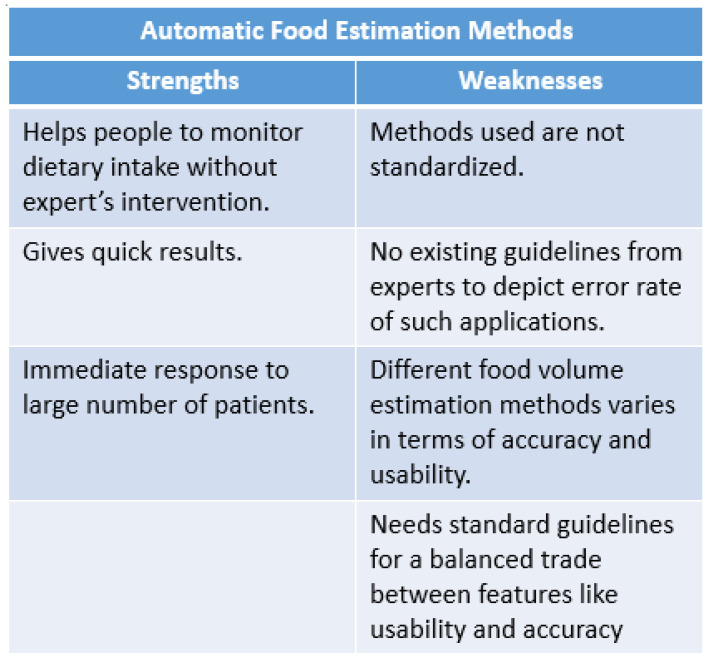
Strengths and weaknesses of automatic food estimation methods.

Automatic food volume estimation method helps people to monitor their dietary intake suffering from chronic diseases without any expert intervention. It gives a quick result as compared to the traditional method which generally involves sending food images to the dietitian. The traditional method involves continuous involvement of dietitians, which makes it unworkable for dietitians to immediately respond to a large number of patients. Conversely, automatic food volume estimation is not standardized, as there are no existing guidelines by experts that refer to the error rate of these applications. Furthermore, different volume estimation methods vary in terms of accuracy and usability. Most of these methods are classified into two categories: single-image-view method and multiple-image-view method. Single-view-image methods are more user friendly, but their accuracy is compromised compared to multiple image view methods as it requires images from different. Therefore, standard guidelines are required for food volume estimation, which should include criteria for a balanced trade between features such as usability and accuracy, and developed applications must be verified according to the standard guidelines. Figure 7 summarizes the strengths and weaknesses of food volume estimation methods.

## 8. Existing and Potential Applications of Vision-Based Methods for Food Recognition in Healthcare

We summarized the core applications of vision-based methods for food recognition in the context of public policy and health care.

### 8.1. mHealth Apps for Dietary Assessment

**Figure 8:**
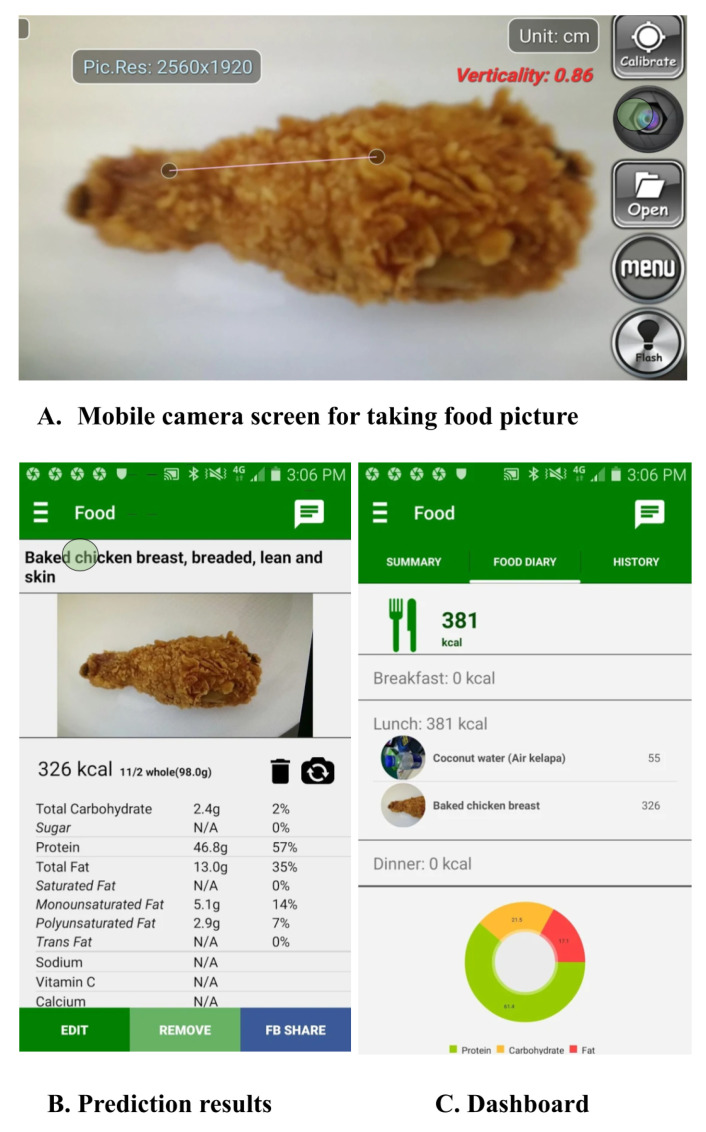
The application provides the top prediction result. This picture is taken from the study of Ghalib et al., 2020 (permission has been obtained from original author).

Today, several mobile applications have been developed to monitor diet and help users to choose healthier alternatives regarding food consumption. Initially, these mobile applications were dependent on manually inputting food items by selecting from limited food databases. Therefore, such applications were not very reliable as they were prone to inaccuracies in dietary assessment, mainly extending from limited exposure to numerous food categories. With the advancement in the area of food image recognition, a large number of mHealth applications for dietary assessment use images to recognize food categories. For this purpose, existing mobile applications use different combinations of traditional and deep visual feature extraction, and classification methods for food recognition described earlier in Section 3 and Section 4. Aizawa et al. [149] developed a mobile app food log, which uses traditional feature-extraction methods such as color, Bag of Features, and SIFT and uses an Adaboost classifier for classification purposes. Similarly, Ravi et al. [150] proposed the ‘FoodCam’ application, which uses traditional methods for feature extraction (LBP and RGB color features) and SVM for classification. Alternatively, Meyers et al. [13] employed a deep visual technique (GoogleNet CNN model) for feature extraction and classification purposes. Similarly, the Food Tracker app proposed by Jiang et al. [151] uses a deep convolutional neural network for feature extraction and classification. Furthermore, G. A. Tahir and C. K. Loo [52] utilized deep visual methods such as ResNet-50, DenseNet201, and InceptionResNet-V2 for feature extraction and Adaptive Reduced Class Incremental Kernel Extreme Learning Machine (ARCIKELM) as a classification method for their mobile application “My Diet Cam”. Table 9 summarizes existing mobile applications in terms of feature extraction and classification methods used. Based on these deep visual method combinations, food recognition accuracies differ for various existing mobile applications. Therefore, apps with higher food recognition and classification accuracies gain more popularity. These apps tend to ease the dietary assessment process. Figure 8 shows the mobile application by Ravi et al. [150].

### 8.2. Harnessing Vision-Based Method to Measure Nutrient Intake during COVID-19

As the COVID-19 is a leading global challenge across the world, maintaining good nutritional status is mandatory for keeping good health to fight against the virus. Automatic vision-based methods for volume estimation and food image recognition in these nutrition tracking apps can assist patients in objectively measuring the nutrient intake of vital vitamins required for boosting the immune system.

### 8.3. Life’s Simple 7

Life’s Simple 7 health score is recently introduced based on modifiable health factors that contribute to heart health. Physical activity, non-smoking status, healthy diet, and body mass index are four modifiable health behaviors in this score. The other three modifiable factors are biological. They include blood pressure, fasting glucose, and cholesterol details. Besides cardiovascular health, Life’s Simple 7 also relates to other health conditions such as venous thromboembolism, cognitive health, atherosclerosis, etc. As dietary intake plays a vital role in computing Life’s Simple 7, manually measuring these factors and then calculating a Life’s Simple 7 score is a very tedious process. This makes it very difficult for both middle-aged patients and elderly patients to keep track of their health. So vision-based methods can play an important role in automating the diet score. However, there are no current studies that have explored this research direction.

### 8.4. Enforcing Eating Ban on Public Places during COVID-19 Pandemic or Other Restricted Places

Vision-based food recognition can automate the enforcement of an eating ban at public places by automatically detecting foods from CCTV and wearable cameras to curb the spread of the virus. Similarly, vision-based food recognition coupled with CCTV or wearable cameras and smart apps automate the enforcement of eating bans at workplaces, laboratories, etc.

### 8.5. Monitoring Malnutrition in Low-Income Countries

Coupling vision-based methods with wearable cameras can automatically detect foods from egocentric images with reasonable accuracy while reducing the burden of processing big data and addressing the user’s privacy concerns. Egocentric images acquired from these cameras are important to study diet and lifestyle, especially in low-income countries with a high malnutrition rate. For example, Jia et al. [157] focused on gathering image data from wearable cameras and discriminating between food/non-food classes based on their tag from the CNN to study human diets. Similarly, Chen et al. [158] studied malnutrition in low- and middle-income countries by using the wearable device e-button.

### 8.6. Food Image Analysis from Social Media

We are in the era of social media, and food is a basic necessity of life, a great deal of content on social media platforms is related to food items. User’s of these platforms frequently share new recipes, new methods of cooking, food pictures after restaurant check-in. Researchers have exploited this data on social media platforms for analyzing dietary intake. For example, Mejova et al. [159] studied food images from foursquare and Instagram to analyze the food consumption pattern in the USA. Similarly, food images on social media platforms are of different cultures. These images can be crawled and then combined together to prepare a large food database.

### 8.7. Food Quality Assessment

Evaluating fruit quality and freshness at the marketplace and at the user end is of increasing interest as opposed to accessing quality at the time of manufacturing. Efforts to date have focused on accessing the quality of foods using vision-based methods. For example, Ismail et al. have contributed an Apple-NDDA dataset [160] that consists of defective and non-defective apple images for food quality assessment.

## 9. Statistical Analysis

**Figure 9:**
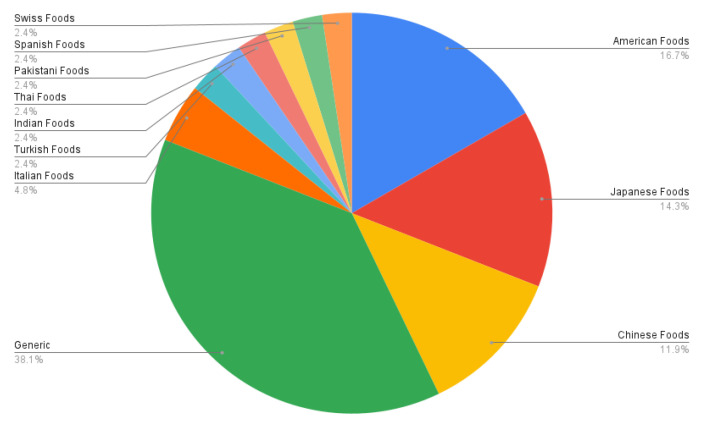
Percentage of datasets summarized according to the types of food. Generic refers to the multi-cultural dataset.

**Figure 10:**
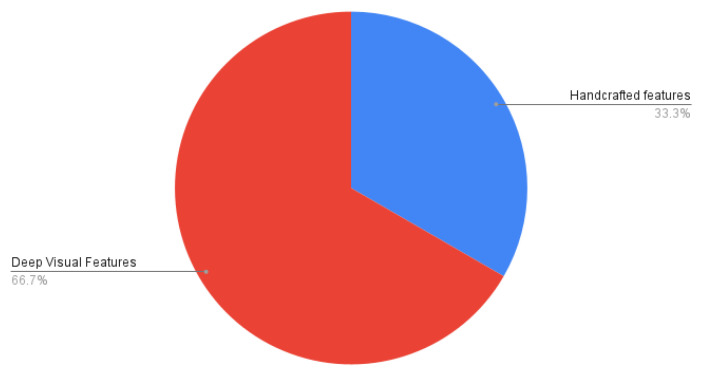
Percentage of studies summarized according to the type of feature extraction methods.

**Figure 11:**
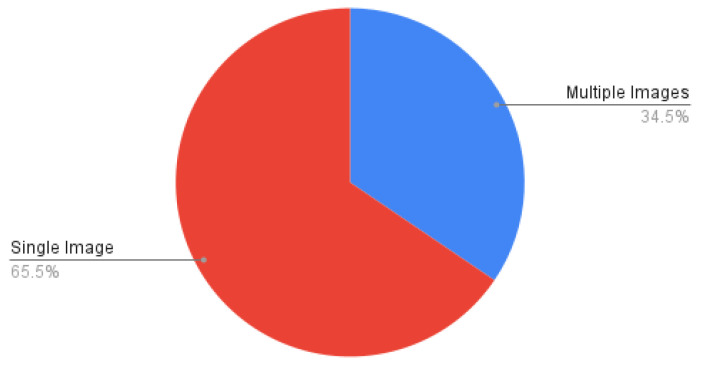
Volume estimation methods using single images vs. multiple images.

**Figure 12:**
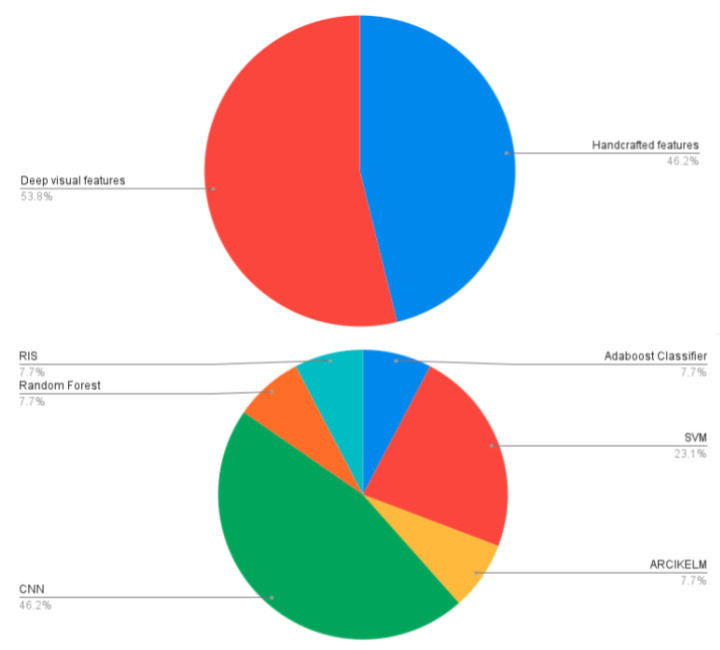
Percentage of studies summarized according to the type of methods employed for feature extraction from food images and the category of classifier used for food image analysis in a mobile application.

We provide a statistical analysis of our study based on the articles and conference proceedings gathered to write this survey paper. We surveyed research studies up to 2020 from various reputed sources: IEEE, Elsevier, ACM, and Web of Sciences. Figure 9 shows a pie chart of the distribution of surveyed food databases according to the country to which the food dishes belong. In it, generic databases are those that contain food dishes of multiple countries. We summarized the surveyed studies in two main categories: studies using handcrafted features, and studies using visual feature representation from convolutional neural networks (CNN), as shown in Figure 10. As discussed in Section 7, volume estimation methods require a single view or multiple images from different viewpoints. We presented a pie chart as shown in Figure 11 that describes the percentage of studies we surveyed according to the number of image viewpoints required to estimate food volume. For ingredient detection, all included studies used CNN due to recent interest in this extension. Similarly, for studies that have implemented mobile applications, the piechart in Figure 12 shows that 46.2% of applications implement CNN for food recognition while remaining mobile applications from surveyed studies are implementing traditional methods for feature extraction.

## 10. Open Issues

This study highlighted open issues based on the survey papers and the authors’ first-hand experience with existing methodologies.

### 10.1. Unsupervised Learning from Unlabelled Dataset

Preparing a large comprehensive annotated data is still a challenge, as manually annotating a dataset is a difficult task with many challenges. Due to the large variety of food dishes, different styles of preparation, etc., it is difficult for an expert dietician to correctly label all the foods, especially in the preparation of a multi-culture food database. Similarly, it involves high costs and a large number of working hours to prepare such a dataset. Recent advancements in contrastive learning have opened a new research paradigm of unsupervised learning. Methods based on contrastive learning such as SimCLR [161] and SwAV [162] do not require labeled datasets and seem to be interesting potential areas of research that future works in food recognition should exploit.

### 10.2. Continual Learning

Food datasets are open-ended, and there is no cap on the number of dishes. So the network must adapt to continuously evolving datasets. All of these properties of food datasets have made them a strong use case for continual learning methods. One of the principal challenges in continuous learning methods is catastrophic forgetting. Catastrophic forgetting refers to completely or abruptly forgetting previously learned information while learning new classes. Many neural networks are susceptible to forgetting during continual learning. It is a prime hindrance in achieving the objective of continuously evolving networks similarly to those of humans. Hence, researchers should also study catastrophic forgetting in the context of food databases.

### 10.3. Explainability

Although there have been numerous attempts, including activation methods, SHAP values [163], and distillation methods, there is still a research gap in the context of food recognition. As food recognition has many domain-specific challenges such as intraclass variations, and non-rigid structure, visualization of the reasoning behind model predictions is vital to trust its decisions. Recently, unsupervised clustering methods [164] are exploited to explain model predictions by distilling knowledge into surrogate models. They provide similar images to test images for explaining prediction results. Explaining prediction results by showing images similar to test images seems more friendly as users do not need any specific domain knowledge to understand these results.

## 11. Discussion

Our research provides deep insight into computer vision-based approaches for dietary assessment. It focuses on both traditional and deep learning methodologies for feature extraction and classification methods used for food image recognition and single- and multi-view methods for volume estimation. Similarly, this survey also explores and compares current food image datasets in detail, as vision-based techniques are highly dependent on a comprehensive collection of food images. In contrast to previous research work, such as work by Mohammad A. Sobhi et al. [165], Min, Weiqing, et al. [166], our survey scrutinizes traditional and current deep visual approaches for feature extraction and classification to enhance clarity in terms of their performance and feasibility. Unlike existing surveys, our survey emphasizes existing solutions developed for food ingredient recognition through multi-label learning. We also reviewed existing computer-based food volume estimation methods in detail, as they have reduced dietitians’ and experts’ intervention and can accurately determine the portion size of the food in contrast to the self-estimation. Finally, our research study also explores real-world applications using the prior methodologies for dietary assessment purposes.

### 11.1. Findings

Our findings indicate that the ultimate performance of traditional and deep visual techniques depends on the type of dataset used. This has been observed from the datasets included from the studies explored in this survey (as shown in Table 1); the three most commonly used datasets were UECFOOD-256 [43], UECFOOD-100 [42], and Food-101 [59]. UECFOOD-256 (25,088 images and 256 classes) and UECFOOD-100 (14,361 images and 100 classes of food) are Japanese food datasets consisting of Japanese food images captured by users, whereas Food-101(101,000 images and 101 classes) is an American fast food dataset containing images crawled from several websites. However, these widely used datasets are region-specific. Therefore, there is an immense need for generic food datasets for excluding regional bias from experimental results. In addition, it is also evident from this survey that deep visual techniques have replaced traditional machine learning methodologies for food image recognition. As per our survey, systems proposed after the year 2015 mainly use deep learning technologies for food classification purposes. This is due to their phenomenal classification performance. In the context of classification performance of deep visual techniques, for food–non-food classification, McAllister et al., 2018 [108] (99.4%), and Pouladzadeh et al., 2016 [104] (99%), achieved the highest top 1 classification accuracy. Pouladzadeh et al., 2016 [104], used DCNN and Graph cut on their proposed dataset, whereas McAllister et al., 2018 [108], used CNN, ANN, SVM, and random forest on the food 5k dataset. Table 5 further compares classification accuracies of proposed deep visual models. Recent advancements and exceptional performance of food image classification methods have now led researchers to explore food images from a much deeper perspective in terms of retrieval and classification of food ingredients from food images. Therefore, we have also explored several proposed solutions for food ingredient recognition and classification. According to our survey, the system proposed by Chen et al., 2016 [47], has achieved the highest F1 score, i.e., 95.88% macro-F1 and 82.06% micro-F1, using the Arch-D method on the UECFOOD-100 dataset (as shown in Table 6). Similarly, automatic food volume estimation methods have reduced dietitians’ and experts’ intervention and can accurately determine the portion size of the food in contrast to the self-estimation for food volume estimation. Single-view methods involve capturing a single image, while multi-views require multiple images to determine accurate food volumes. The results in Table 8 show that multi-view methods are mostly better than single-view methods.

Finally, food category recognition, ingredient classification, and volume estimation techniques helped provide an automatic dietary assessment with reduced human intervention in mHealth apps. For this purpose, we have also surveyed several mobile applications that employ deep learning methods for dietary assessment.

### 11.2. Limitations and Future Research Challenges

Despite enhanced performance and classification accuracy, food image recognition and volume estimation through vision-based approaches may continue to present interesting future research challenges. This is because the performance of the methodologies used for food image identification is highly dependent on the source of images in a particular food dataset. Although a growing number of food categories are being incorporated into food image datasets such as UECFOOD-256 [43], Food 85 [49], and Food201-segmented [13], there is still an immense need for generalized, comprehensive datasets for better performance evaluation and benchmarking. Moreover, we observed that datasets with a large number of food images significantly positively impact classification accuracy. However, keeping these large image datasets updated is another challenge, especially since different types of foods are being prepared every day.

In addition to this, progressive learning during the classification phase is vital for food image datasets due to the continuous arrival of new concepts and domain variation within existing concepts. Similarly, developing frameworks interpretable by highlighting the contribution of the area of interest will improve the overall human trust level on a solution in a real-world environment.

Following food recognition, food volume estimation is a particularly complex and challenging assignment since food items have large variations in shape, texture, and appearances. Our article categorized food portion estimation methods into single-view and multi-view methods. Multi-view methods are more accurate; however, most of these methods also require calibration objects each time and images from different viewpoints, which makes the usability of these solutions tedious for elderly users.

Finally, there is a need to design and develop solutions that can respond to situations ethically. In our context, this refers to the removal of any biases concerning region-specific food preferences. It will help to ensure transparency in existing models.

## 12. Conclusions

In this work, we explored a broad spectrum of vision-based methods that are specifically tailored for food image recognition and volume estimation. In practice, the food recognition process incorporates four tasks: acquiring food images from the corresponding food datasets, feature extraction using handcrafted or deep visual, selection of relevant extracted features, and finally, appropriate selection of classification technique using either traditional machine learning approach or deep learning models followed by food ingredient classification to provide better insight of nutrient information. The findings of surveyed studies have shown that 38.1% of datasets are generic, which includes multi-cultural food dishes. Similarly, 46.2% of surveyed applications implemented CNN for food recognition, while 45.2% of mobile applications have implemented traditional methods for feature extraction. For ingredient detection, several studies used CNN due to its superior performance and recent interest. In addition, 34.5% of techniques for volume estimation require multiple images, while the remaining methods used a single image to estimate food volume.

Despite impeccable performance exhibited by state-of-the-art approaches, there exist several limitations and challenges. There is an immense need for comprehensive datasets for benchmarking and performance evaluation of these models, as incorporating large food image datasets improves the overall performance. Consequently, when dealing with open-ended and dynamic food datasets, the classifier must be capable of open-ended continuous learning. However, existing methods have several bottlenecks, which undermine the food-recognition ability when it comes to open-ended learning, as proposed methods are prone to catastrophic forgetting. They tend to forget previous knowledge extracted from images while learning new information. Such methods work well only for fixed food image datasets. Moreover, our findings indicate that proposed techniques for food ingredient classification still struggle with performance issues when applied to prepared and mixed food items. Survey findings further indicate that CNN models employed for visual feature extraction require labeled datasets for fine-tuning and training. Preparing a labeled food dataset is a difficult task due to the large variety of food dishes. To tackle this problem, unsupervised methods based on contrastive learning seem to have good research potential.

Similarly, automatic food portion estimation methods are categorized into two major categories: single-view-image methods and multi-view-image methods. As discussed earlier, most of multi-view image methods are more accurate than single view methods, but multi-view-image methods require complex processing and images from different angles, resulting in a reduced user retention rate. Furthermore, most of the single and multi-view methods require calibration objects each time, which has made the usability of these solutions tedious for elderly patients.

Therefore, there is substantial room for innovative health care and dietary assessment applications that can integrate wearable devices with a smartphone to revolutionize this research area. Moreover, dietary assessment systems should address these challenges to provide better insights into effective health maintenance and chronic disease prevention. 

## Figures and Tables

**Table 1:** Food image datasets.

Authors	Year	Dataset	Food Category	Total # Images/Class	Image Source
S. Godwin et al. [56]	2006	Wedge Shape foods dataset	American Foods	3 categories	Controlled environment
Chen et al. [44]	2009	PFID	American Fast Foods	1038(61)	Fast food data captured in multiple restaurants
Mariappan et al. [57]	2009	TADA	Artificial And Generic Food	256(11)	Controlled environment
Yanai et al. [49]	2010	Food-50	Japanese Foods	5000(50)	Crawled from web
Hoashi et al. [49]	2010	Food-85	Japanese Foods	8500(85)	Existing food databases
Miyazaki et al. [29]	2011	Foodlog	Japanese Foods	6512(2000)	Captured by users
Marc Bosch et al. [58]	2011	FNDDS	American Foods	7000	Images of food accquired by users
Matsuda et al. [42]	2012	UECFOOD-100	Japanese Foods	14,361(100)	Captured by mobile camera
Chen et al. [48]	2012	ChineseFoodNet	Chinese dishes.	192,000(208)	Gathered from web
M.-Y. Chen et al. [48]	2012	Chen	Chinese Foods	5000/50	Crawled from the Internet
Bossard et al. [59]	2014	Food-101	American Foods	101,000(101)	Crawled from web
L. Bossard et al. [59]	2014	ETHZ Food-101	American Foods	100,000(101)	Crawled from web
Kawano et al. [43]	2014	UECFOOD-256	Japanese Foods	25,088(256)	Captured by mobile camera
T. Stutz et al. [60]	2014	Rice dataset	Generic (Rice)	1 food type	Acquired from user
Farinella et al. [46]	2014	UNCIT-FD889	Italian Foods	3583 (899)	Acquired with a smartphone
Meyers et al. [13]	2015	FOOD201-Segmented	American Foods	12625	Manually annotated dataset
Xin Wang et al. [61]	2015	UPMC Food-101	Generic	100,000(101)	Crawled from web
Cioccoa et al. [50]	2015	UNIMB 2015	Generic	2000(15)	Using a Samsung Galaxy S3 smartphone
Shaobo Fang et al. [62]	2015	TADA(19 foods)	American Foods	19 categories	Controlled environment
Xu et al. [63]	2015	Dishes	Chinese Restaurant Foods	117,504(3832)	Download from dianping
Beijbom et al. [64]	2015	Menu-Match	Generic Restaurant Food	646(41)	Captured from social media
Zhou et al. [65]	2016	Food-975	Chinese Foods	37,785(975)	Collected from restaurants
J. chen et al. [47]	2016	Vireo-Food 172	Chinese Foods	110,241(172)	Downloaded from web
Cioccoa et al. [66]	2016	UNIMB 2016	Italian Foods	1027(73)	Captured from dining tables
Hui Wu et al. [67]	2016	Food500	Generic	148,408(508)	Crawled from web
Singla et al. [68]	2016	Food-11	Generic	16,643(11)	Other food datasets
Farinella et al. [45]	2016	UNCIT-FD1200	Generic	4754(1200)	Acquired using smartphone
Jaclyn Rich et al. [69]	2016	Instagram 800k	Generic	808,964(43)	Social Media
Liang et al. [70]	2017	ECUSTFD	Generic	2978(19)	Acquired using smartphone
Güngör et al. [51]	2017	Turkish-Foods-15	Turkish Dishes	7500/15	Collected from other datasets
Pandey et al. [71]	2017	Indian Food Database	Indian Foods	5000(50)	Downloaded from web
Termritthikun et al. [72]	2017	THFood-50	Thai Foods	700/50	Downloaded from web
Ciocca et al. [73]	2017	FOOD524DB	Generic	247,636(524)	Existing food database
Hou et al. [53]	2017	VegFru	Generic (Fruit and VEG)	160,731(292)	Collected from search engine
Waltner et al. [55]	2017	FruitVeg-81	Generic (Fruit and VEG)	15,630(81)	Collected using mobile phone
Muresan et al. [54]	2018	Generic (Fruits 360 Dataset)	Fruit Dataset	71,125(103)	Camera
Qing Yu et al. [74]	2018	FLD-469	Japanese Foods	209,700(469)	Smart Phone camera
Kaur et al. [75]	2019	FoodX-251	Generic	158,000(251)	Collected from web
Ghalib et al. [52]	2020	Pakistani Food Dataset	Pakistani Dishes	4928(100)	Crawled from web
Narayanan et al. [76]		AI-Crowd	Swiss Foods	25,389	Volunteer Users
Bolaños M. et al. [77]	2016	EgocentricFood	Generic	5038(9)	Taken by a wearable egocentric vision camera
E. Aguilar et al. [78]	2019	MAFood-121	Spanish Foods	21,175	Google search engine

**Table 2:** Handcrafted features.

Reference	Year	Visual Features	Dataset	Recognition Type
Hoashi et al. [49]	2010	Bag-of-features (BoF), Color histogram, Gabor features, and gradient histogram with Multiple Kernel learning.	Used for recognition of 85 food categories	Automatic food recognition
Yang et al. [80]	2010	Deals with pair wise statistics between local features	Pittsburgh Food Image Dataset (PFID)	Food recognition
kong and Tan [86]	2011	SIFT, Guassian Region detector	Pittsburgh Food Image Dataset (PFID) and dataset consisting of food images collected from local restaurants.	Regular shaped foods recognition
Bosh et al. [85]	2011	Global feature classes: texture and color Local features: local entropy color, local color, Garbor filter, SIFT, Haar, Daisy descriptor, Steerable filters and Tamura perceptual filter	Database consisting of food images collected under controlled conditions, from nutritional studies conducted at Prudue University [58]	Food recognition and quantification
Zhang et al. [87]	2011	Color, SIFT, Shape, RGB histograms	Dataset came from online sources, which includes three types of cuisines, two dishes per cuisines were represented by 76 images	Classification of cuisines
Matsuda et al. [88]	2012	Gabor texture features, Histogram of Oriented Gradient (HoG), Bag-of-features of SIFT and CSFIT with Spatial pyramid.	Food image dataset containing 100 different food categories.	Multiple food images recognition
Kawano and Yanai [82]	2013	Bag-of-features and color histogram, HOG patch descriptor and color patch descriptor.	-	Mobile food recognition
Anthimopoulos et al. [89]	2014	Bag-of-features, SIFT and HSV color space	Visual dataset consisting of 5000 food images organized into 11 different classes	Food recognition system for diabetic patients
Tammachat and Pantuwong [90]	2014	Bag-of-features (BoF), Texture and Color	Database consisting of 40 types of Thai food consisting of 100 images of each food type.	Food image recognition
Pouladzadeh et al. [91]	2014	Graph cut, Color and Texture	Dataset consisting of 15 different categories of fruits and food.	Food image recognition for calorie estimation
He et al. [92]	2014	Color, Texture, Dominant Color Descriptor (DCD), Scalable Color Descriptor (SCD), SIFT, Multi-scale Dense SIFT (MDSIFT), Entropy-Based Categorization and Fractal Dimension Estimation (EFD) and Gabor-Based Image Decomposition and Fractal Dimension Estimation (GFD)	Food image dataset containing 1453 images	Food image analysis
Kawano and Yanai [43]	2014	Color, HoG and Fisher Vector	UECFOOD-256 food image dataset	Real-time food image recognition
Oliveira et al. [83]	2014	Color, Texture	Images were gathered using mobile’s camera	Mobile Food Recognition
Pouladzadeh et al. [81]	2015	Color, Texture, Size, Shape, Gabor filter	System was tested on single food portions consisting of fruits and single piece of food. 100 images were chosen for training and 100 for testing purposes.	Cloud-based food recognition.
Farinella et al. [45]	2016	SIFT, Bag of Textons, PRICoLBP	UNICT-FD1200 dataset.	Food image recognition

**Table 3:** Deep visual features.

Reference	Year	Features	Dataset	Recognition Type
Kawano and Yanai, [102]	2014	Fisher Vector and DCNN	UECFOOD-100 and 100-class food Dataset	Food image recognition
Yanai and Kawano, [96]	2015	DCNN	UECFOOD-100 and UECFOOD- 256	Food image recognition
Christodoulidis et al. [103]	2015	CNN	Manually annotated dataset with 573 food items	Food recognition
Pouladzadeh et al. [104]	2016	Graphcut and DCNN	Database consisting of 10,000 high res images	Food recognition for calorie measurement
Hassannejad et al. [105]	2016	Inception	Food-101, UECFOOD-100 and UECFOOD-256	Food image recognition
Liu et al. [106]	2016	DCNN	Food-101, UECFOOD-256	Mobile food image recognition
Chen and Ngo, [47]	2016	Arch-D	Chinese Foods	Ingredient recognition and food categorization
Ciocca et al. [66]	2017	VGG	UNIMIB 2016	Food recognition
Termritthikun et al. [72]	2017	NU-InNet	THFOOD-50	Food recognition
Pandey et al. [71]	2017	AlexNet, GoogLeNet and ResNet	ETH Food-101 and Indian Food Image Database	Food Recognition
Liu et al. [107]	2018	GoogleNet	UECFOOD-100, UECFOOD-256 and Food-101	Food recognition for dietary assessment
McAllister et al. [108]	2018	ResNet-152, GoogLeNet	Food 5k, Food-11, RawFooT-DB and Food-101	Food recognition
Martinel et al. [109]	2018	WISeR	UECFOOD-100, UECFOOD-256 and Food-101	Food recognition
E. Aguilar et al. [110]	2018	AlexNet	UNIMIB2016	Automatic food tray analysis
S. Horiguchi et al. [111]	2018	GoogleNet	Built their own food dataset FoodLog	Food image recognition
Gianluigi Ciocca et al. [112]	2018	ResNet50	Food 475	Food image recognition and classification
B. Mandal et al. [113]	2019	SSGAN	ETH Food-101 and Indian Food Dataset	Food Recognition of Partially Labeled Data
G.Ciocca et al. [114]	2020	GoogleNet, Inception-v3, MobileNet-V2 and ResNet-50	Own dataset containing 20 different food categories of fruit and vegetables.	Food category recognition, Food state recognition
L. Jiang et al. [115]	2020	VGGNet	UECFOOD-100, UECFOOD-256 and introduced new dataset based on FOOD-101.	Food recognition and dietary assesment
C. Liu et al. [116]	2020	VGGNet, ResNet	Vireo-Food 172	Food ingredient recognition
H. Liang et al. [117]	2020		ChineseFoodNet and Vireo-Food 172	Chinese food recognition
H. Zhao et al. [118]	2020	VGGNet, ResNet and DenseNet	UECFOOD-256 and Food-101	Mobile food recognition
G. A. Tahir and C. K. Loo [52]	2020	ResNet-50, DenseNet201 and InceptionResNet-V2	Pakistani Food Dataset, UECFOOD-100, UECFOOD-256, FOOD-101 and PFID	Food recognition
C. S. Won [119]	2020	ResNet50	UECFOOD-256, Food-101 and Vireo-Food 172	Fine grained Food image recognition
Zhidong Shen et al. [120]	2020	Inception-v3, Inception-v4	Dataset was created including hundreds and thousands of images of several food categories	Food recognition and nutrition estimation

**Table 4:** Traditional machine learning methods for food category classification.

Reference	Year	Classification Technique	Classification Accuracy	
			Top 1	Top 5
Hoashi et al. [49]	2010	Multiple Kernel Learning (MKL)	Own Food Dataset = 62.5%	N/A
Yang et al. [80]	2010	Support Vector Machine (SVM)	PFID = 78.0%	N/A
Kong and Tan [86]	2011	Multi-class SVM	PFID = 84%	N/A
Bosh et al. [85]	2011	Support Vector Machine (SVM)	Dataset collected = 86.1% using nutritional studies Conducted at Prudue University	N/A
Zhang et al. [87]	2011	SVM regression with RBF kernel	Own Food Dataset = 82.9%	N/A
Matsuda et al. [88]	2012	Multiple Kernel Learning (MKL) and Support Vector Machine (SVM)	Own food Dataset = 55.8%	N/A
Kawano and Yanai [82]	2013	Linear SVM and fast tookernel	N/A	81.6%
Anthimopoulos et al. [89]	2014	Linear SVM	Own Food Dataset = 78.0%	N/A
Tammachat and Pantuwong [90]	2014	Support Vector Machine (SVM)	Own Food Dataset = 70.0%	N/A
Pouladzadeh et al. [91]	2014	Support Vector Machine (SVM)	Own Food Dataset = 95%	N/A
He et al. [92]	2014	K-nearest Neighbors and Vocabulary Trees	Own Food Dataset = 64.5%	N/A
Kawano and Yanai [43]	2014	One-vs-rest	UECFOOD-256 = 50.1%	UECFOOD-256 = 74.4%
Oliveira et al. [83]	2014	Support Vector Machine (SVM)	Own Food Dataset Top 3 classification achieved between 84 and 100%	N/A
Pouladzadeh et al. [81]	2015	Cloud-based Support Vector Machine	Own Food Dataset = 94.5%	N/A
Farinella et al. [45]	2016	Support Vector Machine (SVM)	UNICT-FD1200 = 75.74%	UNICT-FD1200 = 85.68%

**Table 5:** Deep learning models for food category classification.

Reference	Year	Classification Technique	Classification Performance	
			Top 1	Top 5
Yanai and Kawano [96]	2015	DCNN	UECFOOD-100 = 78.8% UECFOOD-256 = 67.6%	N/A
Christodoulidis et al. [103]	2015	DCNN	Own dataset = 84.9%	N/A
Chen and Ngo [47]	2016	DCNN		
Pouladzadeh et al. [104]	2016	DCNN + Graph cut	Own dataset = 99%	N/A
Hassannejad et al. [105]	2016	DCNN	ETH Food-101 = 88.3% UECFOOD-100 = 81.5% UECFOOD-256 = 76.2%	ETH Food-101 = 96.9% UECFOOD-100 = 97.3% UECFOOD-256 = 92.6%
Liu et al. [106]	2016	CNN	UECFOOD-100 = 76.3% Food-101 = 77.4%	UECFOOD-100 = 94.6% Food-101 = 93.7%
Pandey et al. [71]	2017	Ensemble Net	ETH-Food101 = 72.1% Indian Food = 73.5% Database	ETH-Food101 = 91.6% Indian Food = 94.4% Database
Ciocca et al. [66]	2017	CNN	UNIMIB 2016 = 78.3%	N/A
Termritthikun et al. [72]	2017	CNN	THFOOD-50 = 69.8%	THFOOD-50 = 92.3%
McAllister et al. [108]	2018	CNN+ANN+SVM+ Random Forest	Food-5K = 99.4% Food-11 = 91.3% RawFooT-DB = 99.3% Food-101 = 65.0%	N/A
Liu et al. [107]	2018	DCNN	UECFOOD-256 = 54.5% UECFOOD-100 = 77.5% Food 101 = 77.0%	UECFOOD-256 = 81.8% UECFOOD-100 = 95.2% Food 101 = 94.0%
Martinel et al. [109]	2018	DNN	UECFOOD-100 = 89.6% UECFOOD-256 = 83.2% Food-101 = 90.3%	UECFOOD-100 = 99.2% UECFOOD-256 = 95.5% Food-101 = 98.7%
E. Aguilar et al. [110]	2018	CNN+SVM	UNIMIB 2016 = 90.0%	N/A
Gianluigi Ciocca et al. [112]	2018	CNN	Food-475 = 81.6%	Food-475 = 95.5%
S. Horiguchi et al. [111]	2018	Sequential Personalized Classifier (SPC) with fixed-class and incremental classification	FoodLog = 40.2% (t251-t300)	FoodLog = 56.6% (t251-t300)
B. Mandal et al. [113]	2019	Generative Adversarial Network	ETH Food-101 = 75.3% IndianFood Database = 85.3%	ETH Food-101 = 93.3% Indian Food Database = 95.6%
Aguilar-Torres et al. [122]	2019	CNN based on ResNet-50	MAFood-121 = 81.62%	N/A
Kaiz Merchant and Yash Pande [123]	2019	Inception V3	ETHZ Food-101 = 70.0%	N/A
Mezgec, S. et al. [124]	2019	Deep Learning	Own Food dataset = 93%	N/A
L. Jiang et al. [115]	2020	DCNN (Faster R-CNN)	FOOD20-with-bbx = 71.7%	FOOD20-with-bbx = 93.1%
C. Liu et al., 2020 [116]				
H. Zhao et al. [118]	2020	JDNet	UECFOOD-256 = 84.0% FOOD-101 = 91.2%	UECFOOD-256 = 96.2% FOOD-101 = 98.8%
G. A. Tahir and C. K. Loo [52]	2020	Adaptive Reduced Class Incremental Kernel Extreme Learning Machine (ARCIKELM)	Food-101 = 87.3% UECFOOD-100 = 88.7% UECFOOD-256= 76.51% PFID = 100% Pakistani Food = 74.8%	N/A
C. S. Won [119]	2020	Three-scale CNN	UECFOOD-256 = 74.1% Food 101 = 88.8% Vireo-Food 172 = 91.3%	UECFOOD-256 = 93.2% Food-101 = 98.1% Vireo-Food 172 = 98.9%
Zhidong Shen et al. [120]	2020	CNN	Own dataset = 85.0%	N/A
Jiangpeng He et al. [125]	2020	18 layer ResNet	Own dataset = 88.67%	N/A
Eduardo Aguilar et al. [126]	2020	CNN	Own dataset = 88.67%	N/A
Dario Ortega Anderez et al. [127]	2020	CNN	Own dataset = 97.10%	N/A
G. Song et al. [128]	2020	CNN	Web crawled dataset = 56.47%	Web crawled dataset = 60.33
Limei Xiao et al. [129]	2021	CNN	Own dataset = 97.42%	N/A
Lixi Deng et al. [130]	2021	ResNet-50	School lunch dataset = 95.3%	N/A

**Table 6:** Proposed methods for food ingredient classification.

Reference	Year	Dataset	Method	Recall	Precision	F1
Chen et al. [47]	2016	Vireo-Food 172	Arch-D (Multi-task)	-	-	67.17% (Micro-F1) 47.18% (Macro-F1)
		UECFOOD-100	Arch-D (Multi-task)	-	-	82.06% (Micro-F1) 95.88% (Macro-F1)
Bolaños et al. [131]	2017	Food-101	ResNet50+ Ingredients 101	73.45%	88.11%	80.11%
		Recipe 5k	ResNet50+ Recipe 5k	19.57%	38.93%	26.05%
		Recipe 5k	Inception-v3+ Recipe 5k (Simplified)	42.77%	53.43%	47.51%
Wang, Yunan, et al. [132]	2019	Economic Rice	Inception-V4 + NS (multi-scale)	71.90%	72.10%	71.40%
		Economic Behoon	Inception-V4 + NS (multi-scale)	77.60%	68.50%	69.70%
Salvador, Amaia, et al. [133]	2019	Recipe 1M	CNN Auto-Encoder	75.47%	77.13%	48.61%
J. Chen et al. [135]	2021	VireoFood-172	DCNN	-	-	75.77% (Micro-F1)

**Table 7:** Comparison of single-view methods for food volume estimation.

Reference	Year	Dataset	Results (E: Error%)	Technique
S. Fang [62]	2015	19 food items	E: <6%	3D parameters and reference objects to compute density for estimating the weight of food item
Y. He [36]	2013	1453 food images	E: 11% (beverages) 63%	“Integrated image segmentation and identification system”
T. Miyazaki [29]	2011	6512 images	E: 40%	Linear estimation
Beijbom, O [64]	2015	646 images, with 1386 tagged food items across 41 categories	E: 232 ± 7.2	Restaurant-specific food recognition considers meal as a whole entry with all of its nutrients details in DB to solve the volume estimation problem for the restaurant scenario.
Koichi Okamoto [31]	2016	20 kinds of Japanese Foods (60 test image)	E: 21.30%	Single-image-based food calorie estimation system which uses reference objects to determine food region and quadratic curve estimation from the 2D size of foods to their calories
Pettitt, C [136]	2016	Test data from N:6 participants who completed food diary during pilot sudy by wear micro camera	E: 34%	Wearable micro camera in conjunction with food dairies
Akpa Akpro Hippocrate [34]	2016	119 food images	E: 6.87%	Image processing with cutlery
Jia, W. Y [35]	2012	224 pictures	E: <10%	3D location of a circular feature from a 2D image
Yang, Y. Q [33]	2011	72 images	E: −3.55%	Single digital image, plate reference
Huang, J [39]	2015	fruits (n:6)		imaging processing
Yue, Y [41]	2012	6 food replicas	E: Length (−1.18)	A mathematical model based system involves a camera, circular object in a 3D space to compute food volume.
Zhang, W [38]	2015	15 different kinds of foods	85%	Portion estimation by counting pixels
Rob Comber [137]	2016	6 different meals	“Beef (*E*: −13.89 g, σ: 5.10 g), scrambled egg (*E*: −9.11 g, σ: 8.29 g), Jam sponge (*E*: −12.31 g, σ: 7.03 g) and fish pie (*E*: −12.59 g, σ: 5.74 g). Mean: −9.58”	Visual Assessment
S. Fang [30]	2016	10 objects		“3D geometric models and depth images.”
Godwin, S. [56]	2006	Five portions of 9-inch cake, Seven portions of pizza, Pies were 9 or 10 inches	E: 25%	Estimated portion sizes using a ruler and the adjustable wedge
Hernández, Teresita [37]	2006	101 subjects, 5 foods	E: 4.8% ± 1.8%	Digital photographs printed onto a poster.
Yang et al. [138]	2021	Virtual Food Dataset and Real Food Dataset (RFD) (1500 images)	E: <9% on VFD, E: 11.6% and 20.1% on RFD.	Estimates volume by computing inner product between the probability vector from modified MobileNetV2 and the reference volume vector.
Graikos et al. [139]	2021	EPIC-KITCHENS and their own food video datasets	46.32% average MAPE on 16 test foods and 36.90% average MAPE on 6 combined meals.	Generate 3-dimensional point cloud by using depth map, segmentation mask and camera parameters. It then approximates the volume with points cloud-to-volume algorithm.
Lo, F.P.W et al. [140]	2019	Test dataset: 11 food items	E: 15.32%.	3D point cloud completion from RGB and depth images.

**Table 8:** Comparison of multi-view methods for food volume estimation.

Reference	Year	Dataset	Results (E: Error%)	Technique
F. Zhu [141]	2010	3000 images	E: 1% 19 food items (97.2%)	“Camera calibration step and a 3D volume reconstruction step”
Xu Chang [141]	2013	14 to 20 images for multi-view method	E: 7.4% to 57.3%	Multi-view volume estimation using “Shape from Silhouettes” to estimate the food portion size
Kong, Fanyu [12]	2015	6 food items	84–91%	Multi-View RGB images for 3D reconstruction to estimate the volume
Trevno, Roberto [142]	2015	120 students (n = 120 meals; 57 breakfast + 63 lunch)	74% (reliability)	Digital Food Imaging Analysis (DFIA)
Jia, W. Y [143]	2014	100 food samples	E: −2.80% 30%	ebutton is used for taking pictures, and then portion size is calculated semi-automatically by using computer software
Xu, C [36]	2013		E: 10%	3D MODELLING AND POSE ESTIMATION
Rhyner, D [144]	2016	6 meals	85.10%	Multi-View RGB images, reference card and 3D model for volume estimation
T. Stutz [60]	2014	Rice, blinded servings	E: <33%	Mobile Augmented Reality System
Makhsous et al. [145]	2020	8 food items tested	40% improvement in the accuracy of volume estimation as compared to manual calculation.	Employs a mobile Structured Light System (SLS) to measure the food volume and portion size of a dietary intake.
Yuan et al. [146]	2021	Test dataset: 6 food items	E: 0.83 5.23%.	3D reconstruction from multi-view RGB images.
Lo, F.P.W et al. [140]	2019	Test dataset: 11 food items	E: 15.32%.	3D point cloud completion from RGB and depth images.

**Table 9:** Summary of feature extraction and classification methods used by existing mobile applications.

Reference	Year	Application Name	Food Segmentation	Feature Extraction Method	Classification Method
Aizawa et al. [149]	2013	FoodLog	No	Color, SIFT and Bag of Features	Adaboost Classifier
Oliveira et al. [83]	2014	-	Yes	Color and Texture	Support Vector Machine (SVM)
Probst et al. [152]	2015	-	-	SIFT, LBP and Color	Linear SVM
Meyers et al. [13]	2015	Im2Calories	Yes	GoogleNet CNN	GoogleNet CNN model
Ravi et al. [150]	2015	FoodCam	No	HoG, LBP and RGB Color Features	Linear SVM
Waltner et al. [55]	2017	-	Yes	RGB, HSV and LAB Color values	Random Forest Classifier
Mezgec and Seljak [153]	2017	-	-	NutriNet	NutriNet
Pouladzadeh et al. [154]	2017	-	Yes	CNN	Caffe Framework
Waltner et al. [155]	2017	-	Yes	CNN	CNN
Ming et al. [11]	2018	DietLens	-	ResNet-50 CNN	ResNet-50 CNN
Jiang et al. [151]	2018	-	Yes	Colors, Lines, Points, SIFTand Texture Features	Reverse Image Search (RIS) and Text Mining
Jianing Sun et al. [156]	2019	Food Tracker	Yes	DCNN	DCNN
G. A. Tahir and C.K. Loo [52]	2020	MyDietCam	Yes	ResNet-50, DenseNet201 and Inception ResNet-V2	Adaptive Reduced Class Incremental Kernel Extreme Learning Machine (ARCIKELM)

## Data Availability

Not applicable.
